# Central and Branch Retinal Artery Occlusion—Do They Harbor the Same Risk of Further Ischemic Events?

**DOI:** 10.3390/jcm10143093

**Published:** 2021-07-13

**Authors:** Joanna Roskal-Wałek, Paweł Wałek, Michał Biskup, Dominik Odrobina, Jerzy Mackiewicz, Stanisław Głuszek, Beata Wożakowska-Kapłon

**Affiliations:** 1 Collegium Medicum, The Jan Kochanowski University, 25-317 Kielce, Poland; pwalek@ujk.edu.pl (P.W.); magdale_5@hotmail.com (D.O.); stanislaw.gluszek@ujk.edu.pl (S.G.); bw.kaplon@poczta.onet.pl (B.W.-K.); 2 Ophthalmology Clinic, Voivodeship Regional Hospital, 25-736 Kielce, Poland; michal.biskup@onet.pl; 3 1st Clinic of Cardiology and Electrotherapy, Swietokrzyskie Cardiology Centre, 25-736 Kielce, Poland; 4 Ophthalmology Clinic Boni Fratres Lodziensis, 93-357 Łódź, Poland; 5 Department of Vitreoretinal Surgery, Medical University of Lublin, 20-079 Lublin, Poland; jerzymackiewicz@umlub.pl; 6 Oncological, Endocrinological and General Surgery Clinic, Voivodeship Regional Hospital, 25-736 Kielce, Poland

**Keywords:** retinal artery occlusion, stroke, myocardial infarction, all-cause mortality

## Abstract

Purpose: Retinal artery occlusion (RAO) is associated with an increased risk of cardiovascular events such as ischemic stroke and myocardial infarction, but whether different RAO subtypes such as central retinal artery occlusion (CRAO) or branch retinal artery occlusion (BRAO) carry similar risk of these events is unclear. Our aim was to determine whether the risk of cardiovascular events differs between CRAO and BRAO. Methods: This single-center, retrospective study included 131 patients hospitalized in our clinic in 2010–2020 with CRAO or BRAO confirmed by ophthalmic examination. Data on demographics, previous ischemic stroke and myocardial infarction, comorbidities, the results of echocardiographic and ultrasound carotid artery examinations and laboratory tests were assessed. Data on ischemic stroke, myocardial infarction, and all-cause mortality occurring after RAO were obtained from the Polish National Health Service, which collects data on all publicly funded hospitalizations. Using these data, Kaplan-Meier analyses and Cox proportional hazard regression were performed. Results: Ischemic stroke occurred in 9.9% of patients after RAO: 10.6% in the CRAO group and 8.1% in the BRAO group (*p* = 0.662). Myocardial infarction occurred in 2.3% of patients after RAO: 2.1% in the CRAO group and 2.7% in the BRAO group (*p* = 0.843). All-cause mortality occurred in 22.9% of patients after RAO: 25.5% in the CRAO group and 16.2% in the BRAO group (*p* = 0.253). The composite endpoint of ischemic stroke, myocardial infarction, and all-cause mortality after RAO occurred in 28.2% of patients: 30.9% in the CRAO group and 21.6% in the BRAO group (*p* = 0.338). There was no difference between CRAO and BRAO in median time to ischemic stroke (32 vs. 76.4 months; *p* = 0.352), all-cause mortality (35.9 vs. 36.3 months; *p* = 0.876) or composite endpoint (37.5 vs. 41.5 months; *p* = 0.912) after RAO. The Kaplan-Meier analysis showed no differences between CRAO and BRAO in ischemic stroke, myocardial infarction, all-cause mortality, or the composite endpoint; similar results were obtained in analyses of patients with and without cardiovascular events before RAO. Conclusions: The prognosis for ischemic stroke, myocardial infarction, and all-cause mortality is similar in patients with CRAO and BRAO. Ischemic strokes occur with a similar frequency before and after RAO. Myocardial infarctions are observed significantly more frequently before an episode of RAO than after. The results of our study indicate that both CRAO and BRAO require expanded diagnostics to assess the risk of recurrent cardiovascular events, especially ischemic strokes, to implement appropriate prophylaxis and reduce mortality.

## 1. Introduction

**Figure 1:**
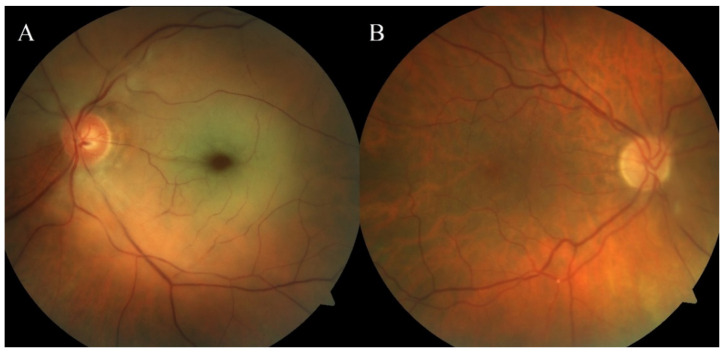
Fundus images of CRAO (**A**) and BRAO (**B**). Figure 1A shows CRAO in the left eye with diffuse retinal whitening due to ischemia and cherry-red spot. Figure 1B shows BRAO in the right eye with visible emboli in a retinal artery.

Retinal artery occlusion (RAO) is an emergency state that requires urgent diagnosis and treatment [1,2,3,4]. Depending on which type of vessel is affected, RAO can be divided into central retinal artery occlusion (CRAO) and branch retinal artery occlusion (BRAO) (Figure 1) [4,5]. Despite their low incidence rates in the general population, CRAO and BRAO have important clinical implications [1,2,3,4,6]. Both CRAO and BRAO are the ocular equivalent of a cerebral infarction [7] and have similar etiology and risk factors to ischemic stroke [1,2,3,4,6]. RAO is most commonly the consequence of an embolism from the ipsilateral carotid artery, heart, or aortic arch, which leads to partial or complete occlusion of the central retinal artery or its branches; however, some differences in the etiology of CRAO and BRAO have been noted [1,2,3,4,8,9,10,11,12].

The characteristic risk factors for CRAO and BRAO are nearly identical to those for ischemic stroke, with hypertension being the most common risk factor for both CRAO and BRAO [1,2,3,4,6,10,11,12,13,14,15]. Similar to patients with stroke, patients with RAO are at high risk of subsequent cardiovascular events [6,15,16,17,18,19,20,21,22,23,24,25,26,27,28,29,30,31,32,33,34,35]. Risk of stroke after CRAO and BRAO is significantly higher than in control groups [6,25], but whether this risk is the same for CRAO and BRAO is unclear [6,12,15,18,35,36]. Nevertheless, the impact of RAO on the risk of further ischemic stroke is still a matter of debate, especially in reference to the incidence rate of stroke after RAO [1,3,37,38]. Risk of myocardial infarction after RAO is also higher than in control groups, but stratification of this risk by RAO subtype has been done in only one study so far, and this study was conducted in an Asian population [23]. It should be borne in mind that Asian populations differ from European populations and the results of such studies should be interpreted with caution [29,39]. In Europe only one longitudinal prospective study has assessed the risk of stroke, myocardial infarction, and death after RAO, but CRAO and BRAO were not compared [22]. Recently, several studies from Europe have assessed the risk of stroke in patients with CRAO and BRAO, but the results vary [12,17,35]. So far, the risk of stroke, myocardial infarction, and death has not been compared between patients with CRAO and BRAO in a European population.

In addition to guidelines, which recommend quick brain imaging and diagnostic workup in patients with acute RAO, it is also crucial to stratify risk of cardiovascular events according to RAO subtype [3]. Moreover, cardiovascular events are the leading cause of death in Europe [40]; it is therefore necessary to understand the relationship between these incidents and both CRAO and BRAO.

In this study we assessed and compared the risk of ischemic stroke, myocardial infarction, and all-cause mortality in European patients with CRAO and BRAO.

## 2. Materials and Methods

### 2.1. Database

The study protocol was approved by the Ethics Committee of Jan Kochanowski University in Kielce, Poland. The study included 131 patients hospitalized in 2010–2020 at the Department of Ophthalmology of the Provincial Integrated Hospital in Kielce due to an ophthalmologically confirmed RAO. Diagnosis of CRAO and BRAO was based on eye fundus examination by an ophthalmologist. Additional examinations were performed in uncertain cases. The dates of ischemic stroke, myocardial infarction, and all-cause mortality were determined using data obtained from the Polish National Health Fund. Almost every Polish citizen is covered by medical care guaranteed by the National Health Fund, which also collects data on dates of and reasons for hospitalization, and procedures performed during hospitalization. Data regarding the causes of hospitalization are coded using the International Classification of Diseases 10 (ICD-10) system. Each Polish citizen has a personal identity called a Universal Electronic System for Registration of the Population number, which allows the patient to be identified in the healthcare system.

### 2.2. Study Sample

This retrospective study included all inpatients of the Department of Ophthalmology treated for RAO (ICD-10: H34.1, H34.9) in the years 2010–2020. Initially, 134 events encoded as RAOs were included. Following verification, one RAO record was excluded because it was a recurrent RAO and the analysis included only first RAO events. Recurrent RAO was included in the analysis as a stroke event according to the current American Heart Association/American Stroke Association guidelines [7]. One patient was excluded due to elevated inflammatory parameters that could indicate Horton’s disease, and one patient was excluded due to retina changes that might indicate Susac syndrome. Finally, 131 patients with confirmed RAO were included in the analysis. The date of the cardiovascular event was considered to be the first day of hospitalization due to ischemic stroke or myocardial infarction. Hospitalizations encoded with I63, I63.0, I63.1, I63.2, I63.3, I63.4, I63.5, I63.8, and I63.9 codes according to ICD-10 were considered stroke cases. Hospitalizations due to myocardial infarction were identified by I21, I21.0, I21.2, I21.3, I21.4, and I21.9 codes. Date of all-cause mortality was determined using data from the National Health Fund. Each patient was tracked for up to 11 years from the time of RAO diagnosis.

Data on sex, age, body-mass index (BMI), and blood pressure at the time of admission to the Department of Ophthalmology, concomitant diseases, and the results of imaging and laboratory tests were obtained from medical records of the hospitalization for RAO. Carotid ultrasound examination was performed in 84.7% of patients and echocardiography in 89.3% of patients. Lipid profile analysis was conducted in 85.5% of patients. Creatinine levels were assessed and creatinine clearance estimated using the simplified Modification of Diet in Renal Disease (MDRD) formula in 83.2% of patients. Patients were classified as having hypercholesterolemia if they were diagnosed with hypercholesterolemia before admission, had elevated total cholesterol (>190 mg/dL) or low-density lipoprotein (LDL)-cholesterol levels on admission, or were using lipid-lowering drugs at the time of RAO. Patients were classified as having ischemic heart disease if they had a history of myocardial infarction, had percutaneous coronary intervention or coronary artery bypass grafting, or were diagnosed with ischemic heart disease by a cardiologist. Atherosclerotic plaque was diagnosed by a radiologist with carotid ultrasound examination. Chronic kidney disease stage was determined using the MDRD calculation based on creatinine levels during hospitalization for RAO.

### 2.3. Statistical Analysis

Results were presented as means ± standard deviations (SDs) or counts and percentages. The demographic and clinical characteristics of patients with CRAO were compared to those of patients with BRAO using a student’s *t*-test for normally distributed variables and a Mann-Whitney test or Chi-squared test for non-normally distributed variables. Times to event were presented as medians and interquartile ranges (IQR) and compared using the Mann-Whitney U-test. The composite endpoint was defined as the occurrence of ischemic stroke, myocardial infarction, or all-cause mortality. The stroke-free rate, myocardial infarction-free rate, all-cause mortality-free rate, and composite endpoint-free rate were estimated using the Kaplan-Meier method and log-rank tests were used to describe and compare the curves of patients with CRAO and BRAO. Kaplan-Meier analysis was also performed for patients who had no history of cardiovascular events, such as ischemic stroke or myocardial infarction, before the occurrence of RAO (i.e., patients who did not undergo secondary prevention of recurrent cardiovascular events).

Univariate Cox proportional hazard regression analyses were performed to identify hazards associated with the composite endpoint for CRAO and BRAO. The assumption of hazard proportionality was verified in all analyzed cases and the proportional hazard test was statistically insignificant.

Statistical significance was set at *p* < 0.05. Statistical analyses were performed with STATISTICA 13.3 software (TIBCO Software Inc., Tulsa, OK, USA).

## 3. Results

The characteristics of the study population, divided into CRAO and BRAO groups, are presented in Table 1. Comparative analysis showed no difference between groups in sex, mean age, BMI, comorbidities, or the affected eye. Patients younger than 50 years less frequently had CRAO; however, only six (4.6%) patients in this age group were included in the study population. Significantly more patients with CRAO than BRAO were in the 60–69 years age group (37.2% vs. 18.9%; *p* = 0.043). We found no differences in intima media measurements, or the incidence of carotid stenosis >70%, ipsilateral stenosis >70% to RAO, or ipsilateral carotid occlusion, based on ultrasound results. Atherosclerotic plaque in the carotid arteries was significantly more frequent in patients with CRAO than in those with BRAO (91.3% vs. 72.4%; *p* = 0.012). Analysis of echocardiographic results showed no differences between patients with CRAO and BRAO. Lipid profile analysis showed that triglyceride levels were lower in patients with CRAO than in those with BRAO (139 vs. 186.9 mg/dL; *p* < 0.001). There were no differences in creatinine concentration, MDRD, or fasting glycemia between patients with CRAO and BRAO.

During follow-up, we registered 12 ischemic strokes and one second episode of RAO classified as an ischemic stroke, making a total of 13 strokes (9.9%), as well as three (2.3%) myocardial infarctions, 30 (22.9%) all-cause mortality, and 37 (28.2%) occurrences of the composite endpoint.

Ischemic strokes occurred with a similar frequency before and after RAO (13 vs. 9.9%; *p* = 0.431) but myocardial infarctions were observed significantly more frequently before an episode of RAO than after (25.2 vs. 2.3; *p* < 0.001). There were no differences in the incidence of ischemic stroke before and after CRAO among the CRAO subpopulation (11.7 vs. 10.6%; *p* = 0.811), while myocardial infarction was significantly more frequent before the CRAO episode (25.5 vs. 2.1%; *p* <0.001). Similarly, it was observed in the subpopulation of BRAO patients, where there were no differences in the occurrence of ischemic stroke before and after BRAO (16.2 vs. 8.1%; *p* = 0.286), while myocardial infarction was significantly more frequent before the BRAO episode (24.3 vs. 2.7%; *p* = 0.007).

Over 635.9 persons-years (including 464.6 persons-years for CRAO and 171.3 persons-years for BRAO), the incidence rate of stroke was 20.7 (95% CI 11.5–34.5) per 1000 persons-years for the total study population, 21.7 (95% CI 11–38.7) per 1000 persons-years for CRAO, and 17.9 (95% CI 4.5–48.6) per 1000 persons-years for BRAO (CRAO vs. BRAO; *p* = 0.767). Over 662.6 persons-years (including 487.8 persons-years for CRAO and 174.8 persons-years for BRAO), the incidence rate of myocardial infarction was 4.5 (95% CI 1.2–12.3) per 1000 persons-years for the total study population, 4.1 (95% CI 0.7–12.5) per 1000 persons-years for CRAO, and 5.7 (95% CI 0.3–28.2) per 1000 persons-years for BRAO (CRAO vs. BRAO; *p* = 0.785). Over 570.1 persons-years (including 418.1 persons-years for CRAO and 152 persons-years for BRAO), the incidence rate of all-cause mortality was 52.6 (95% CI 36.2–74.2) per 1000 persons-years for the total study population, 57.4 (95% CI 37.6–84.1) per 1000 persons-years for CRAO, and 39.5 (95% CI 16–81.1) per 1000 persons-years for BRAO (CRAO vs. BRAO; *p* = 0.409). Over 549.3 persons-years (including 406.4 persons-years for CRAO and 142.9 persons-years for BRAO), the incidence rate of the composite endpoint was 67.4 (95% CI 48.2–91.9) per 1000 persons-years for the total study population, 71.4 (95% CI 48.7–101.2) per 1000 persons-years for CRAO, and 56.4 (95% CI 26–106.2) per 1000 persons-years for BRAO (CRAO vs. BRAO; *p* = 0.54) (Table 2).

**Figure 2:**
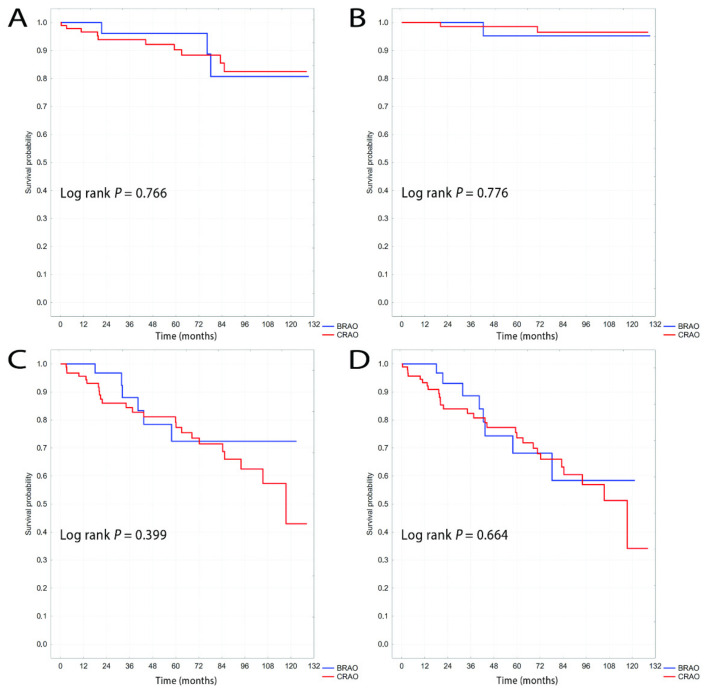
Kaplan-Meier curves of survival probability for entire study population for ischemic stroke (**A**), myocardial infarction (**B**), all-cause mortality (**C**) and the composite endpoint (**D**).

Figure 2 presents the results of the Kaplan-Meier survival curve analysis for the entire study population. The log-rank test showed no statistical differences in the incidence of ischemic stroke, myocardial infarction, all-cause mortality, or the composite endpoint between CRAO and BRAO during the 11-year follow-up. There was a trend towards later occurrence of ischemic stroke in BRAO than in CRAO, but this was not statistically significant.

**Figure 3:**
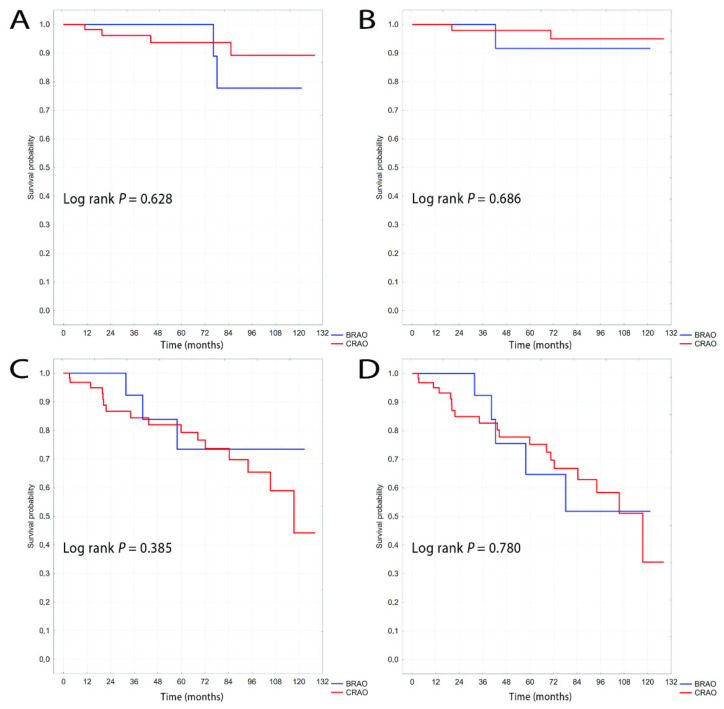
Kaplan-Meier curves of survival probability for subpopulation without cardiovascular events before RAO for ischemic stroke (**A**), myocardial infarction (**B**), all-cause mortality (**C**) and the composite endpoint (**D**).

Figure 3 shows the results of Kaplan-Meier analyses for patients without cardiovascular events such as ischemic stroke or myocardial infarction before RAO (i.e., patients not undergoing secondary prevention of cardiovascular events). This analysis also showed no differences between CRAO and BRAO. There was a trend towards later occurrence of ischemic stroke, deaths, and the composite endpoint in patients with BRAO, but this was not statistically significant over the 11-year follow-up. In our opinion, the later occurrence of events such as stroke, death, or the composite endpoint in those without cardiovascular events before BRAO could be due to the small number of patients with this subtype of RAO in the study population.

Cox proportional hazard regression showed that the risk of the composite endpoint after RAO increased with age for both RAO subtypes, although this did not reach statistical significance (Table 3). The significance threshold was reached only in patients with CRAO aged over 80 years. In some age groups it was not possible to calculate the hazard ratio because the number of events was too low. The risk of the composite endpoint increased with the severity of aortic stenosis in patients with CRAO. Among the laboratory test results, higher levels of total cholesterol and high-density lipoprotein (HDL) -cholesterol significantly reduced the risk of the composite endpoint in patients with CRAO. In patients with BRAO, higher creatinine clearance significantly reduced the risk of the composite endpoint.

## 4. Discussion

In our study, we showed that the prognosis for ischemic stroke, myocardial infarction, and all-cause mortality is similar for CRAO and BRAO over 11 years of follow-up. The risk is similar for both subtypes of RAO, both in patients with and without a history of ischemic stroke or myocardial infarction before RAO. To our knowledge, this is the first study to compare the risk of cardiovascular events and all-cause mortality between CRAO and BRAO in a European population. BRAO, which has a much more favorable prognosis than CRAO in terms of final visual acuity and is caused by smaller size of the emboli, appears to be similar to CRAO in terms of the risk of cardiovascular events and death. The results of our research indicate that patients with BRAO should undergo the same diagnostic workup as patients with CRAO.

Risk factors for atherosclerosis are common in patients with RAO; they are often multiple and undiagnosed [1,2,3,4,6,9,11,13,17]. In studies comparing CRAO and BRAO the incidence of selected cardiovascular risk factors, such as hypertension, diabetes mellitus, dyslipidemia, obesity, smoking, and renal disease was similar in both types of RAO [11,13,15,19]. However, some differences between CRAO and BRAO were found in the incidence of coronary artery disease, valvular heart disease, or atrial fibrillation [11,13]. In our study we did not find significant differences in cardiovascular risk factors between subtypes of RAO, the only significant difference among cardiovascular risk factors was observed in triglyceride levels, which were significantly higher for BRAO than for CRAO. The reason for the higher triglyceride concentration in the BRAO group than in the CRAO group is unclear, although Schilling et al. showed that lesions of small vessels in the central nervous system are more common in patients with hypertriglyceridemia, which may suggest that small vessel disease should be considered in the etiopathogenesis of BRAO [41].

As in other studies, arterial hypertension was the most frequently identified risk factor, found in 84.7% of RAO patients [4,6,10,11,12,13,15,16,17,18,19,20].

### 4.1. Ischemic Stroke

The frequency of observed strokes during our 11-year follow-up was 9.9% for the entire study population. This differs substantially from the results of Rim et al., who identified stroke in 15% of patients with RAO and in 8% of controls during 10 years of follow-up [18]. In the study by Chang et al., which also included transient ischemic attacks (TIA), the percentage of patients who had a stroke after RAO was 19.61% compared with 10.05% in the control group [6]. There may be several reasons for this difference. Firstly, we assessed only the risk of ischemic stroke after RAO, whereas Rim et al. included ischemic, hemorrhagic, and unspecified strokes in their analyses [18]. However in the study by Chang et al., 14% of patients had an ischemic stroke after RAO, which is still a higher proportion than in our study. Moreover, the follow-up period in the study by Chang et al. was 3 years [6]. Secondly, in contrast to the studies by Chang et al. and Rim et al. [6,18] our study focused on a European population and ethnic differences may influence the results of cardiovascular events both in patients with stroke and RAO [29,39,42]. Shaikh et al. found that Black and Asian people, as well as Native Americans, have a higher risk of stroke after RAO than White people [29]. Third, our study is not based solely on a database review, as were the studies by Chang et al. and Rim et al. [6,18]. When using databases, it is possible to include patients incorrectly diagnosed with RAO, which may affect the results.

Recently, Laczynski et al. demonstrated that the risk of stroke after RAO is lower than that reported in previous studies based on large databases [38]. The authors observed that, when all patients diagnosed with RAO using ICD-9 codes were included, 22.5% were diagnosed with stroke. A similar percentage is presented in studies based on large databases [6,18,38]. Revision of medical documentation allowed the diagnosis to be verified. The authors then found that only five patients (2.3%) had a stroke. The authors state that this difference undermines the results of studies based on large databases using ICD-9 codes and indicate that the risk of stroke may be much lower [38]. In a prospective study on a European population conducted by Hankey et al., 10.2% of patients experienced a stroke during the 10-year follow-up, which is similar to the result obtained in our study. However, Hankey et al. did not stratify their results according to RAO subtype [22].

Kaplan-Meier curves from the studies by Rim et al. and Chang et al. show a much more rapid decline in the number of stroke-free patients immediately after RAO than those from the study by Hankey et al. [6,18,22]. In the study by Chang et al. the risk of stroke was highest in the first month after RAO [6]. Such a high risk of stroke immediately after CRAO was also confirmed in the study by Park et al. [28]. This relationship has been demonstrated not only in the Asian population. A similar study by French et al., which analyzed records from the US Medicare database, showed an increased risk of stroke in the time immediately surrounding CRAO [25]. In contrast to these studies, we did not observe any sudden increase in the number of strokes after RAO for either subtype [6,18,25,28].

In the most recent prospective study from Europe, by Leisser et al., diagnosis of stroke or TIA after RAO was based on information obtained from the patient by telephone call 1 year after the RAO. Within 1 year after RAO one patient had a stroke again and another had a TIA. This study showed no significant differences between CRAO and BRAO in the percentage of strokes or TIA before or after RAO [35]. We also did not find a difference between CRAO and BRAO in the incidence of ischemic strokes before and after RAO. The study by Lee et al. also showed no differences in the incidence of previous history of cerebrovascular accident or TIA between patients with CRAO and BRAO, no statistically significant differences were also detected between CRAO and BRAO and the presence of acute ischemic lesions observed in diffusion-weighted MRI within 7 days of the onset of visual symptoms [15]. The studies by Leisser et al. and Lee et al. are based on very small study groups (30 and 33 patients, respectively) but the study by Rim et al., which included 401 patients with RAO, also did not show differences in the frequency of strokes between patients with CRAO and BRAO [15,18,35]. The high percentage of strokes (24.2%, 37.5% of which were without neurological symptoms) found on magnetic resonance imaging (MRI) within 7 days after RAO in the study by Lee et al. is noteworthy [15]. Similar results were also reported in non-Asian populations in studies that assessed ischemic changes in the central nervous system with MRI within 7 days after RAO [12,21]. Most of the observed new ischemic lesions were clinically silent. Since patients did not undergo MRI during hospitalization in our study, we cannot discuss the incidence of silent brain infarcts. However, the recent meta-analysis by Fallico et al. showed no differences in the frequency of symptomatic or asymptomatic strokes between patients with CRAO and BRAO [31]. The study by Kim et al. also showed no significant differences between CRAO and BRAO in terms of lesions on MRI examination, such as the coexistence of stroke, small vessel disease, silent lacunar infarction, cerebral microbleeds, and white matter hyperintensity [11].

Nevertheless, several studies indicate a difference in the incidence of strokes after CRAO and BRAO [6,12,19,36]. In the study by Avery et al., 32.3% of patients with CRAO and 11.4% of patients with BRAO had a stroke before an ocular episode and these patients were excluded from further analysis. 4.8% of patients experienced ischemic stroke after CRAO and 12.9% after BRAO. The incidence of strokes up to 3 years after the diagnosis of retinal ischemia was 35.5% for CRAO and 22.9% for BRAO. The authors suggest that CRAO has higher association with stroke than BRAO, with stroke generally presenting earlier in CRAO than in BRAO [19]. Lauda et al. demonstrated that BRAO but not CRAO is an independent risk factor for symptomatic and silent acute brain infarction. The authors speculate that not only the size and composition of the embolic material but also unrecognized mechanisms, may play a role in the pathophysiology of RAO [12]. Recently, attention has been paid to the role of small vessel disease in the etiology of RAO [10,11]. In the study by Schor et al. the risk of stroke within 1 year after RAO was lower for BRAO than for CRAO and transient RAO [36].

In the study by Chang et al. patients with CRAO had a significantly higher incidence of stroke than those with BRAO and controls during the 3-year follow-up period. The incidence rate ratio was 2.71 (95% CI 1.81–4.06) for CRAO versus controls, 1.97 (95% CI 1.47–2.64) for BRAO versus controls, and 1.95 (95% CI 1.27–3.00) for CRAO versus BRAO. The authors propose that the difference might be attributable to more extensive occlusion of the main blood supply in CRAO [6]. The study by Lee et al. showed that the main causes, including large artery arteriosclerosis and cardiac sources of embolism, were found significantly more often in CRAO than BRAO (38.9% vs. 6.7%; *p* = 0.046), but this did not result in a difference in the frequency of acute cerebral ischemic lesions between CRAO and BRAO [15]. In the study by Kim et al., in respect to etiology only cardioembolism was significantly higher in the CRAO compared to BRAO group (17% vs. 6%; *p* = 0.018) however the type of RAO was not significantly associated with co-incident cerebral infarction [11]. We did not find significant differences in carotid stenosis between patients with CRAO and BRAO, but carotid atherosclerotic plaques were significantly more common in CRAO; however, this did not increase the frequency of ischemic strokes in the CRAO group. We found no statistically significant differences in echocardiography changes between groups. Hayreh et al. did not show significant differences in changes on carotid Doppler ultrasound or echocardiography between patients with CRAO and BRAO [4]. Similarly, the study by Schmidt et al. showed no significant differences in carotid Doppler ultrasound between patients with CRAO and BRAO [13]. Echocardiography and Doppler ultrasound of carotid arteries were recommended in all patients with RAO in both studies [4,13]. The highest risk of early stroke recurrence occurs in patients with a stroke of thrombotic or embolic etiology in the course of large artery atherosclerosis and the most common etiology of RAO is carotid atherosclerosis [1,2,3,4,17,20,43].

### 4.2. Myocardial Infarction

In our study, myocardial infarction after RAO occurred in three patients (2.3%), which differs notably from the results of other studies [16,22]. Only Laczynski et al. showed a relatively low risk of myocardial infarction after RAO (3.6%) [38]. In the study by Hankey et al. 14.3% of patients experienced a myocardial infarction [22]. An even higher percentage of myocardial infarctions after CRAO was seen by Lavin et al. [16]: 17% of patients experienced myocardial infarction within 2 years of follow-up. In the studies by Hankey et al. and Laczynski et al. myocardial infarctions were reported more frequently than strokes after RAO [22,38]. Notably, these studies were conducted in European and American populations [22,38]. In the study by Hayreh & Zimmerman, which was conducted in an American population, there were more myocardial infarctions than TIA/strokes before or after RAO [8]. In our study there were more ischemic strokes than myocardial infarctions after RAO; however, when we summed up myocardial infarctions and ischemic strokes before and after RAO there were more myocardial infarctions than ischemic strokes.

No myocardial infarctions were found in the study by Callizo et al. during 4 weeks of follow-up [17]. In the study by Hong et al. 0.7% of myocardial infarction was reported during 1 year of follow-up [20]. These data suggest that myocardial infarction does not occur as often as ischemic stroke after RAO. These assumptions are confirmed by the study by Park et al., in which 0.9% of patients experienced an myocardial infarction and 8.7% had ischemic stroke in an observation period covering 365 days before and after CRAO [28].

The frequency of myocardial infarction after RAO in our study is similar to the frequency of myocardial infarction reported after ischemic strokes or TIA, which is estimated at 0.49%–1.32% per year after the incident [44].

Only the study by Chang et al. analyzed the relationship between acute coronary syndrome and CRAO and BRAO in detail. Their results showed that acute coronary syndrome occur significantly more often in patients with RAO than in controls (5.4% vs. 3.3%) and more frequently in CRAO than in BRAO (9.6% vs. 4.1%) [23].

### 4.3. Mortality

The results of several studies indicate increased mortality in patients with RAO. The results of the Beaver Dam Eye Study (BDES) and the Blue Mountains Eye Study (BMES) were analyzed to investigate the association between RAO and mortality in older people. During 10 to 12 years of follow-up, the mortality of patients who developed RAO was almost twice as high as that of patients who did not. Cardiovascular mortality was 30% vs. 16%, and mortality due to stroke was 12% vs. 4% in these groups, respectively [33].

In the study by Hankey et al., 29.6% of patients died during the 10-year follow-up. Ischemic heart disease was the cause of more than half of deaths (58.6%), while stroke was the cause of only one death (3.4%) [22].

Our result is similar to that of Hankey et al., although our population was older (mean age 64 vs. 70 years). The study by Hankey et al. was conducted at the turn of the 1970s and 1980s, and the availability of healthcare, modern pharmacotherapy, and interventional treatment for ischemic heart disease have since improved [22]. In our study, all-cause mortality occurred in 22.9% of patients during 11 years of follow-up, which is much more than the 5.4% observed by Laczynski et al. during 14 years of follow-up (2004–2018). The patient population in the study by Laczynski et al. was younger than that in our study (mean 66.1 vs. 70 years) [38]. Our result is similar to that of the study by De Potter & Zografos [30], which, in contrast to the BMES and BDES, did not show a statistically significant survival difference between patients with RAO and age- and sex-matched controls. The study by De Potter & Zografos is the only one to compare survival prognosis between CRAO and BRAO as well as between RAO and controls. A lower survival rate was observed in patients with visible embolus and patients with BRAO than in the control group. Importantly, no such difference was found between CRAO and BRAO [30].

In our study, we did not observe any difference in all-cause mortality between CRAO and BRAO.

### 4.4. Composite Endpoint

To date, several studies have analyzed a composite endpoint of cardiovascular events in patients with RAO [16,20,26,38]. However, no studies have compared a composite endpoint between CRAO and BRAO. In our study, the incidence of a composite endpoint of ischemic stroke, myocardial infarction, and all-cause mortality reached 28.2% for RAO, with no statistically significant differences between CRAO and BRAO. In the study by Mir et al., the composite endpoint, including ischemic and hemorrhagic strokes, TIA, myocardial infarctions, or mortality, was noted in 19% of the study population; however, only events during hospitalization were analyzed [26].

In the study by Lavin et al. the composite endpoint included stroke, myocardial infarction, and death during the 2-year follow-up period was observed in 32% of patients [16]. In the study by Hong et al. the incidence of the composite endpoint, defined as any kind of stroke, myocardial infarction, or cardiovascular death within 1 year, was 9.9%; 57.1% of these events occurred within 30 days after RAO, 21.4% occurred 30–90 days after RAO, and 21.4% occurred 91–365 days after RAO [20]. In the study by Laczynski et al., the composite endpoint, consisting of stroke, myocardial infarction, and death was seen also in 10% of RAO patients but in this study follow-up period was 14 years [38].

### 4.5. Strengths and Limitations

Our study included only patients with a diagnosis of RAO confirmed by our research team. Thus, we eliminated incorrect or doubtful diagnoses. All patients had their medical history records reviewed for confirmation of CRAO or BRAO and two patients were excluded due to suspicion of Horton’s disease and Susac syndrome. Only European patients were included in our study, making the study population homogeneous in terms of ethnicity, which is important due to reported differences in the risk of ischemic stroke and RAO between ethnic groups. The follow-up period for the studied population was 11 years, which places our study among the studies with the longest follow-up after RAO. In our clinic, patients are very carefully examined and analyzed after RAO, which allowed for very detailed information on the characteristics of the study group and a high percentage of echocardiographic examinations, carotid ultrasounds, and additional tests (lipid profile analysis). Patients were monitored for ischemic stroke, myocardial infarction, and all-cause mortality instead of just one selected cardiovascular event, which provides a broad view of cardiovascular risk after RAO.

We did not have detailed data on the cause of death; therefore, we chose all-cause mortality for the analysis, especially as the causes of sudden death are not always verified. Some of the deaths could be caused by undiagnosed ischemic stroke or myocardial infarction, which could reduce the percentage of accumulated cardiovascular events, such as ischemic stroke or fatal myocardial infarction. For this reason, we decided to present analyses for a composite endpoint. The dates of ischemic strokes and myocardial infarctions were obtained from the National Health Fund database using ICD-10 codes; therefore, these diagnoses could not be verified by our team as was done for RAO diagnoses. Patients with BRAO constituted only 28.3% of the study population. This is caused by the mildly symptomatic nature of this type of RAO, which means that some patients do not visit, or are delayed in visiting, the ophthalmologist.

Because social health insurance by the National Health Fund is used mainly in the territory of Poland, there may be a bias resulting from lack of reporting of the analyzed events outside Poland. The region of Poland where the study took place is in the center of the country, so we consider the risk of such a bias to be minimal. The reported differences in the incidence of ischemic stroke and RAO in different ethnic groups do not allow us to generalize our results to other populations.

## 5. Conclusions

The prognosis for ischemic stroke, myocardial infarction, and all-cause mortality is similar for CRAO and BRAO. This relationship is similar for patients with and without cardiovascular events such as ischemic stroke or myocardial infarction before RAO. Ischemic strokes occur with a similar frequency before and after RAO. Myocardial infarctions are observed significantly more frequently before an episode of RAO than after. Patients with CRAO and BRAO exhibit similar risk factors for cardiovascular diseases, the only difference being the concentration of triglycerides, which is higher in patients with BRAO.. The results of our study indicate that both CRAO and BRAO require extended imaging diagnostics such as diffusion-weighted MRI of the central nervous system, carotid Doppler ultrasonography, echocardiography and systemic vascular risk assessment using laboratory evaluations, blood pressure and electrocardiographic monitoring to assess the risk of recurrence of cardiovascular events, especially ischemic strokes, in order to implement appropriate prophylaxis and reduce mortality.

## Figures and Tables

**Table 1:** Characteristics of the study population.

	Total RAO population *n* = 131	CRAO *n* = 94 (71.7%)	BRAO *n* = 37 (28.3%)	*p*-Value
Sex (female), *n* (%)	46 (35.1)	35 (37.2)	11 (29.7)	0.417
Age, years (SD)	70 (11.2)	70.9 (10.1)	67.7 (13.7)	0.449
Age <65 years, *n* (%)	38 (29.5)	27 (29.4)	11 (29.7)	0.909
Age <50 years, *n* (%)	6 (4.6)	2 (2.1)	4 (10.8)	0.032
Age 50–59 years, *n* (%)	13 (9.9)	7 (7.5)	6 (16.2)	0.131
Age 60–69 years, *n* (%)	42 (32.1)	35 (37.2)	7 (18.9)	0.043
Age 70–79 years, *n* (%)	45 (34.4)	31 (33)	14 (37.8)	0.043
Age ≥80 years, *n* (%)	25 (19.1)	19 (20.2)	6 (16.2)	0.600
BMI 18.5–24.9, *n* (%)	37 (28.2)	29 (30.9)	8 (21.6)	0.291
BMI 25–29.9, *n* (%)	51 (38.9)	39 (41.5)	12 (32.4)	0.338
BMI 30–34.9, *n* (%)	34 (26)	22 (23.4)	12 (32.4)	0.289
BMI 35–39.9, *n* (%)	7 (5.3)	(3 (3.2)	4 (10.8)	0.081
BMI ≥40, *n* (%)	2 (1.5)	1 (1.1)	1 (2.7)	0.491
Systolic pressure, mmHg (SD)	139 (23.7)	138 (22.9)	141.6 (25.7)	0.380
Diastolic pressure, mmHg (SD)	81.9 (10.4)	81.6 (11.2)	82.6 (8)	0.582
Hypertension, *n* (%)	111 (84.7)	79 (84)	32 (86.5)	0.726
Hypercholesterolemia, *n* (%)	95 (72.5)	68 (72.3)	27 (73)	0.942
Coronary artery disease, *n* (%)	52 (39.7)	38 (40.4)	14 (37.8)	0.785
Myocardial infarction before RAO, *n* (%)	33 (25.2)	24 (25.5)	9 (24.3)	0.886
Diabetes mellitus, *n* (%)	27 (20.6)	22 (23.4)	5 (13.5)	0.208
Ischemic stroke before RAO, *n* (%)	17 (13)	11 (11.7)	6 (16.2)	0.489
Hemorrhagic stroke before RAO, *n* (%)	3 (2.3)	2 (2.1)	1 (2.7)	0.843
Smoking habits, *n* (%)	32 (24.4)	26 (27.7)	6 (16.2)	0.170
Atrial fibrillation, *n* (%)	19 (14.5)	13 (13.8)	6 (16.2)	0.856
Permanent atrial fibrillation, *n* (%)	9 (6.9)	7 (7.5)	2 (5.4)	0.667
Paroxysmal atrial fibrillation, *n* (%)	10 (7.6)	6 (6.4)	4 (10.8)	0.390
Oral anticoagulant, *n* (%)	17 (13)	12 (12.8)	5 (13.5)	0.909
Acetylsalicylic acid, *n* (%)	30 (22.9)	21 (22.3)	9 (24.3)	0.808
Heart failure, *n* (%)	27 (20.6)	19 (20.2)	8 (21.6)	0.856
Chronic kidney disease (stage)	2.5 (0.7)	2.5 (0.7)	2.4 (0.6)	0.321
Chronic kidney disease, stage 4–5, *n* (%)	4 (3.7)	4 (5.3)	0 (0)	0.176
Chronic kidney disease, stage ≤3, *n* (%)	53(49.1)	39 (52)	14 (42.4)	0.359
RAO of the left eye, *n* (%)	62 (47.3)	45 (47.8)	17 (46)	0.842
RAO of the right eye, *n* (%)	69 (52.7)	49 (52.1)	20 (54.1)	0.836
Ischemic stroke after RAO, *n* (%)	13 (9.9)	10 (10.6)	3 (8.1)	0.662
Myocardial infarction after RAO, *n* (%)	3 (2.3)	2 (2.1)	1 (2.7)	0.843
Death after RAO, *n* (%)	30 (22.9)	24 (25.5)	6 (16.2)	0.253
Composite endpoint after RAO, *n* (%)	37 (28.2)	29 (30.9)	8 (21.6)	0.338
Median time to stroke, months (IQR)	44.4 (57.2)	32 (52.3)	76.4 (56.8)	0.352
Median time to myocardial infarction, months (IQR)	42.5 (50.5)	45.4 (50.5)	-¹	-
Median time to death, months (IQR)	35.9 (43.3)	35.9 (53.7)	36.3 (11.6)	0.876
Median time to composite endpoint, months (IQR)	40.4 (33.8)	37.5 (37.2)	41.5 (18.3)	0.912
Intima media measurement, mm (SD) ^2^	1.2 (0.4)	1.2 (0.4)	1.2 (0.3)	0.844
Atherosclerotic plaque in the carotid arteries, *n* (%) ^3^	94 (86.2)	73 (91.3)	21 (72.4)	0.012
Carotid stenosis >70%, *n* (%) ^4^	19 (17.1)	14 (17.5)	5 (16.1)	0.863
Ipsilateral stenosis to RAO, *n* (%) ^4^	15 (13.5)	12 (15)	3 (9.7)	0.462
Ipsilateral occlusion of the carotid artery, *n* (%) ^4^	4 (3.6)	2 (2.5)	2 (6.5)	0.316
Diameter of the ascending aorta, mm (SD) ^5^	34.1 (4.3)	34.3 (4.3)	33.6 (4.5)	0.477
Ivs., mm (SD) ^5^	11.2 (1.7)	11.2 (1.8)	11.2 (1.6)	0.698
LA, mm (SD) ^5^	39.7 (7)	39.4 (7.1)	40.4 (7)	0.499
LVEF, % (SD) ^5^	54.9 (7.7)	55.2 (7.6)	54.1 (8.1)	0.731
Calcifications on the mitral or aortic valves, *n* (%) ^5^	51 (43.6)	41 (47.7)	10 (32.3)	0.138
Mild aortic stenosis, *n* (%) ^5^	5 (4.2)	5 (5.8)	0 (0)	0.172
Moderate aortic stenosis, *n* (%) ^5^	4 (3.4)	4 (4.7)	0 (0)	0.222
Severe aortic stenosis, *n* (%) ^5^	1 (0.9)	1 (1.2)	0 (0)	0.547
PFO, *n* (%) ^6^	5 (4.3)	2 (2.3)	3 (9.7)	0.083
Total cholesterol, mg/dL (SD)	196.5 (48.4)	194.2 (45.7)	201.8 (54.4)	0.445
LDL-cholesterol, mg/dL (SD)	120.5 (42.2)	119.4 (41.2)	122.9 (44.9)	0.360
HDL-cholesterol, mg/dL (SD)	46.3 (10.3)	47.4 (10.9)	43.6 (8.5)	0.104
Triglycerides, mg/dL (SD)	153.6 (79.4)	139 (45.7)	186.9 (82.7)	<0.001
Creatinine, mg/dL (SD)	1.2 (0.35)	1.2 (0.37)	1.1 (0.3)	0.614
MDRD, mL/min (SD)	61.9 (18.2)	60.6 (18.9)	64.8 (16.4)	0.360
Fasting glucose, mg/dL (SD)	111.2 (33.8)	112 (36.2)	109.4 (27.8)	0.914

BMI, body mass index; BRAO, branch retinal artery occlusion; CRAO, central retinal artery occlusion; HDL, high-density lipoprotein; IQR, interquartile range; Ivs., interventricular septum; LA, left atrial antero-posterior diameter; LDL, low-density lipoprotein; LVEF, left ventricular ejection fraction; MDRD, Modification of Diet in Renal Disease; PFO, patent foramen ovale; RAO, retinal artery occlusion; SD, standard deviation. ^1^ Median time could not be determined as there was only one observation. ^2^ 64.9% of patients had intima media measurements. ^3^ Atherosclerotic plaque was assessed in 109 patients (83% of the studied population). ^4^ The variable was assessed in 111 patients (84.7% of the studied population). ^5^ Echocardiography was performed in 117 patients (89.3% of the studied population). ^6^ PFO was confirmed by transesophageal echocardiography.

**Table 2:** Persons-years and the incident rate for the analyzed events.

		RAO	CRAO	BRAO	*p*-Value CRAO vs. BRAO
Stroke	PY	635.9	464.6	173.3	
	IR	20.7	21.7	17.9	0.767
Myocardial infarction	PY	662.6	487.8	174.8	
	IR	4.5	4.1	5.7	0.785
All-cause mortality	PY	570.1	418.1	152	
	IR	52.6	57.4	39.5	0.409
Composite endpoint	PY	549.3	406.4	142.9	
	IR	67.4	71.4	56.4	0.54

BRAO, branch retinal artery occlusion; CRAO, central retinal artery occlusion; IR, incident rate; PY, persons-years.

**Table 3:** Univariate Cox regression analyses for the composite endpoint for CRAO and BRAO.

	CRAO			BRAO		
	HR	95% CI	*p*-Value	HR	95% CI	*p*-Value
Age	1.04	1–1.08	0.061	1.11	0.99–1.24	0.064
Sex (male)	1.14	0.52–2.49	0.756	0.7	0.14–3.52	0.661
Age <65 years	0.41	0.15–1.07	0.069	-	-	-
Age ≥65 years	2.46	0.93–6.51	0.069	-	-	-
Age 60–69 years	0.61	0.26–1.43	0.252	-	-	-
Age 70–79 years	1.11	0.52–2.39	0.786	3.66	0.73–18.24	0.114
Age ≥80 years	2.43	1.05–5.63	0.039	1.59	0.32–7.94	0.572
BMI 18.5–24.9	2.03	0.94–4.37	0.072	2.48	0.48–12.88	0.281
BMI 25–29.9	0.48	0.21–1.10	0.083	0.64	0.15–2.72	0.549
BMI 30–34.9	0.95	0.40–2.28	0.917	1.08	0.21–5.44	0.930
BMI 35–39.9	3.75	0.48–29.23	0.207	1.37	0.17–11.26	0.768
Systolic pressure	0.99	0.98–1.01	0.710	0.99	0.97–1.02	0.717
Diastolic pressure	4.02	0.98–1.05	0.295	0.99	0.91–1.08	0.850
Hypertension	1.63	0.56–4.72	0.369	-	-	-
Hypercholesterolemia	0.80	0.37–1.74	0.573	0.29	0.07–1.24	0.094
Coronary artery disease	1.43	0.68–3.00	0.348	1.84	0.43–7.77	0.408
Myocardial infarction	1.41	0.59–3.36	0.433	0.71	0.14–3.56	0.680
Smoking habits	0.99	0.44–2.27	0.997	1.09	0.22–5.41	0.918
Diabetes mellitus	1.32	0.56–3.11	0.530	0.82	0.11–7.56	0.942
Ischemic stroke	0.89	0.27–2.96	0.846	0.55	0.07–4.47	0.574
Atrial fibrillation	1.71	0.65–4.53	0.278	0.67	0.08–5.44	0.707
Acetylsalicylic acid	1.47	0.59–3.64	0.410	1.34	0.27–6.69	0.722
Heart failure	1.56	0.69–3.56	0.286	0.97	0.20–4.86	0.975
CKD stage	0.88	0.48–1.59	0.662	2.19	0.57–8.38	0.251
Intima media thickness	1.37	0.56–3.34	0.484	5.05	0.19–133.6	0.332
Atherosclerotic plaque in the carotid arteries	0.82	0.24–2.75	0.746	-	-	-
Carotid stenosis >70%	0.99	0.37–2.67	0.990	2.34	0.25–21.51	0.454
Ipsilateral stenosis to RAO	0.88	0.30–2.58	0.815	7.83	0.70–87.58	0.095
Calcifications on the mitral or aortic valves	1.64	0.70–3.81	0.254	2.27	0.51–10.21	0.284
Mild aortic stenosis	1.05	0.14–7.83	0.961	-	-	-
Moderate aortic stenosis	2.92	0.98–8.68	0.055	-	-	-
Severe aortic stenosis	5.96	0.77–46.26	0.088	-	-	-
PFO	0.68	0.09–5.19	0.710	-	-	-
Total cholesterol	0.99	0.98–0.998	0.023	0.99	0.98–1.01	0.319
LDL-cholesterol	0.99	0.98–1.00	0.183	0.99	0.97–1.01	0.239
HDL-cholesterol	0.94	0.90–0.99	0.020	0.96	0.88–1.04	0.319
Triglycerides	0.99	0.985–1.00	0.095	0.999	0.99–1.01	0.932
Creatinine	0.82	0.25–2.66	0.736	3.40	0.22–52.35	0.381
MDRD	0.98	0.96–1.01	0.208	0.94	0.88–1.01	0.009
Fasting glucose	1.00	0.99–1.01	0.925	0.96	0.91–1.01	0.123

BMI, body mass index; BRAO, branch retinal artery occlusion; CKD, chronic kidney disease; CRAO, central retinal artery occlusion; HDL, high-density lipoprotein; HR, hazard ratio; LDL, low-density lipoprotein; MDRD, Modification of Diet in Renal Disease; PFO, patent foramen ovale.

## Data Availability

The data underlying this article will be shared on reasonable request to the corresponding author.

## References

[1] Dattilo M., Newman N.J., Biousse V. (2018). Acute retinal arterial ischemia. Ann. Eye Sci..

[2] Chen C.S., Varma D., Lee A. (2020). Arterial Occlusions to the Eye: From Retinal Emboli to Ocular Ischemic Syndrome. Asia-Pacific J. Ophthalmol..

[3] Biousse V., Nahab F., Newman N.J. (2018). Management of Acute Retinal Ischemia. Ophthalmology.

[4] Hayreh S.S., Podhajsky P.A., Zimmerman M.B. (2009). Retinal Artery Occlusion: Associated Systemic and Ophthalmic Abnormalities. Ophthalmology.

[5] Hayreh S.S. (2011). Acute retinal arterial occlusive disorders. Prog. Retin. Eye Res..

[6] Chang Y.-S., Jan R.-L., Weng S.-F., Wang J.-J., Chio C.-C., Wei F.-T., Chu C.-C. (2012). Retinal Artery Occlusion and the 3-Year Risk of Stroke in Taiwan: A Nationwide Population-Based Study. Am. J. Ophthalmol..

[7] Sacco R.L., Kasner S.E., Broderick J.P., Caplan L.R., Connors J., Culebras A., Elkind M.S., George M.G., Hamdan A.D., Higashida R.T. (2013). An Updated Definition of Stroke for the 21st Century. Stroke.

[8] Hayreh S.S., Zimmerman M.B. (2017). Ocular Arterial Occlusive Disorders and Carotid Artery Disease. Ophthalmol. Retin..

[9] Wilson L., Warlow C., Russell R. (1979). Cardiovascular Disease in Patients with Retinal Arterial Occlusion. Lancet.

[10] Cho K.H., Kim C.K., Woo S.J., Park K.H., Park S.J. (2016). Cerebral Small Vessel Disease in Branch Retinal Artery Occlusion. Investig. Opthalmolo. Vis. Sci..

[11] Kim Y.D., Kim J.Y., Park Y.J., Park S.J., Baik S.H., Kang J., Jung C., Woo S.J. (2021). Cerebral magnetic resonance imaging of coincidental infarction and small vessel disease in retinal artery occlusion. Sci. Rep..

[12] Lauda F., Neugebauer H., Reiber L., Jüttler E. (2015). Acute Silent Brain Infarction in Monocular Visual Loss of Ischemic Origin. Cerebrovasc. Dis..

[13] Schmidt D., Hetzel A., Geibel-Zehender A., Schulte-Mönting J. (2007). Systemic diseases in non-inflammatory branch and central retinal artery occlusion--an overview of 416 patients. Eur. J. Med. Res..

[14] O’Donnell M.J., Xavier D., Liu L., Zhang H., Chin S.L., Rao-Melacini P., Rangarajan S., Islam S., Pais P., McQueen M.J. (2010). Risk factors for ischaemic and intracerebral haemorrhagic stroke in 22 countries (the INTERSTROKE study): A case-control study. Lancet.

[15] Lee J., Kim S.W., Lee S.C., Kwon O.W., Kim Y.D., Byeon S.H. (2014). Co-occurrence of Acute Retinal Artery Occlusion and Acute Ischemic Stroke: Diffusion-Weighted Magnetic Resonance Imaging Study. Am. J. Ophthalmol..

[16] Lavin P., Patrylo M., Hollar M., Espaillat K.B., Kirshner H., Schrag M. (2018). Stroke Risk and Risk Factors in Patients with Central Retinal Artery Occlusion. Am. J. Ophthalmol..

[17] Callizo J., Feltgen N., Pantenburg S., Wolf A., Neubauer A.S., Jurklies B., Wachter R., Schmoor C., Schumacher M., Junker B. (2015). Cardiovascular Risk Factors in Central Retinal Artery Occlusion. Ophthalmology.

[18] Rim T.H., Han J., Choi Y.S., Hwang S.-S., Lee C., Lee S.C., Kim S.S. (2016). Retinal Artery Occlusion and the Risk of Stroke Development. Stroke.

[19] Avery M.B., Magal I., Kherani A., Mitha A.P. (2019). Risk of Stroke in Patients with Ocular Arterial Occlusive Disorders: A Retrospective Canadian Study. J. Am. Hear. Assoc..

[20] Hong J.-H., Sohn S.-I., Kwak J., Yoo J., Ahn S.J., Woo S.J., Jung C., Yum K.S., Bae H.-J., Chang J.Y. (2017). Retinal artery occlusion and associated recurrent vascular risk with underlying etiologies. PLoS ONE.

[21] Helenius J., Arsava E.M., Goldstein J.N., Cestari D.M., Buonanno F.S., Rosen B.R., Ay H. (2012). Concurrent acute brain infarcts in patients with monocular visual loss. Ann. Neurol..

[22] Hankey G., Slattery J.M., Warlow C.P. (1991). Prognosis and prognostic factors of retinal infarction: A prospective cohort study. BMJ.

[23] Chang Y.-S., Chu C.-C., Weng S.-F., Chang C., Wang J.-J., Jan R.-L. (2014). The risk of acute coronary syndrome after retinal artery occlusion: A population-based cohort study. Br. J. Ophthalmol..

[24] Deijle I.A., Van Schaik S.M., Van Wegen E.E., Weinstein H.C., Kwakkel G., Berg-Vos R.M.V.D. (2017). Lifestyle Interventions to Prevent Cardiovascular Events After Stroke and Transient Ischemic Attack. Stroke.

[25] French D.D., Margo C.E., Greenberg P.B. (2018). Ischemic Stroke Risk in Medicare Beneficiaries with Central Retinal Artery Occlusion: A Retrospective Cohort Study. Ophthalmol. Ther..

[26] Mir T.A., Arham A.Z., Fang W., Alqahtani F., Alkhouli M., Gallo J., Hinkle D.M. (2019). Acute Vascular Ischemic Events in Patients With Central Retinal Artery Occlusion in the United States: A Nationwide Study 2003-2014. Am. J. Ophthalmol..

[27] Chodnicki K.D., Pulido J.S., Hodge D.O., Klaas J.P., Chen J.J. (2019). Stroke Risk Before and After Central Retinal Artery Occlusion in a US Cohort. Mayo Clin. Proc..

[28] Park S.J., Choi N.-K., Yang B.R., Park K.H., Lee J., Jung S.-Y., Woo S.J. (2015). Risk and Risk Periods for Stroke and Acute Myocardial Infarction in Patients with Central Retinal Artery Occlusion. Ophthalmology.

[29] Shaikh I.S., Elsamna S.T., Zarbin M.A., Bhagat N. (2020). Assessing the risk of stroke development following retinal artery occlusion. J. Stroke Cerebrovasc. Dis..

[30] De Potter P., Zografos L. (1993). Survival prognosis of patients with retinal artery occlusion and associated carotid artery disease. Graefe’s Arch. Clin. Exp. Ophthalmol..

[31] Fallico M., Lotery A.J., Longo A., Avitabile T., Bonfiglio V., Russo A., Murabito P., Palmucci S., Pulvirenti A., Reibaldi M. (2020). Risk of acute stroke in patients with retinal artery occlusion: A systematic review and meta-analysis. Eye.

[32] Zhou Y., Zhu W., Wang C. (2016). Relationship between retinal vascular occlusions and incident cerebrovascular diseases. Medicine.

[33] Wang J.J., Cugati S., Knudtson M.D., Rochtchina E., Klein R., Klein B.E., Wong T.Y., Mitchell P. (2006). Retinal Arteriolar Emboli and Long-Term Mortality. Stroke.

[34] Klein R., Klein B.E.K., Moss S.E., Meuer S.M. (2003). Retinal Emboli and Cardiovascular Disease. Arch. Ophthalmol..

[35] Leisser C., Findl O. (2019). Rate of strokes 1 year after retinal artery occlusion with analysis of risk groups. Eur. J. Ophthalmol..

[36] Schorr E.M., Rossi K., Stein L.K., Park B.L., Tuhrim S., Dhamoon M.S. (2020). Characteristics and Outcomes of Retinal Artery Occlusion. Stroke.

[37] Hayreh S.S. (2018). Do Patients With Retinal Artery Occlusion Need Urgent Neurologic Evaluation?. Am. J. Ophthalmol..

[38] Laczynski D.J., Gallop J., Lyden S.P., Bena J., Yuan A., Smolock C.J., Caputo F.J. (2020). Retinal artery occlusion does not portend an increased risk of stroke. J. Vasc. Surg..

[39] Wolma J., Nederkoorn P.J., Goossens A., Vergouwen M.D.I., Van Schaik I.N., Vermeulen M. (2009). Ethnicity a risk factor? The relation between ethnicity and large- and small-vessel disease in White people, Black people, and Asians within a hospital-based population. Eur. J. Neurol..

[40] Nichols M., Townsend N., Luengo-Fernandez R., Leal J., Gray A., Scarborough P. (2012). European Cardiovascular Disease Statistics. European Heart Network, Brussels, European Society of Cardiology, Sophia Antipolis. https://www.escardio.org/static-file/Escardio/Press-media/press-releases/2013/EU-cardiovascular-disease-statistics-2012.pdf.

[41] Schilling S., Tzourio C., Dufouil C., Zhu Y., Berr C., Alpérovitch A., Crivello F., Mazoyer B., Debette S. (2014). Plasma lipids and cerebral small vessel disease. Neurology.

[42] . Ahuja R.M., Chaturvedi S., Eliott D., Joshi N., Puklin J.E., Abrams G.W. (1999). Mechanisms of retinal arterial occlusive disease in African American and Caucasian patients. Stroke.

[43] Hankey G.J. (2003). Long-Term Outcome after Ischaemic Stroke/Transient Ischaemic Attack. Cerebrovasc. Dis..

[44] Boulanger M., Béjot Y., Rothwell P.M., Touzé E. (2018). Long-Term Risk of Myocardial Infarction Compared to Recurrent Stroke After Transient Ischemic Attack and Ischemic Stroke: Systematic Review and Meta-Analysis. J. Am. Heart Assoc..

